# Health systems’ preparedness to provide post-abortion care: assessment of health facilities in Burkina Faso, Kenya and Nigeria

**DOI:** 10.1186/s12913-022-07873-y

**Published:** 2022-04-22

**Authors:** Kenneth Juma, Ramatou Ouedraogo, Joshua Amo-Adjei, Ali Sie, Mamadou Ouattara, Nkechi Emma-Echiegu, Joseph Eton, Michael Mutua, Martin Bangha

**Affiliations:** 1 African Population and Health Research Center, P.O. Box 10787, Manga Cl, Nairobi, Kenya; 2 University of Cape Coast, Cape Coast, Ghana; 3 Centre de Recherche en Santé de Nouna, Ouagadougou, Burkina Faso; 4 Ebonyi State University, Abakaliki, Nigeria; 5 University of the Witwatersrand, Johannesburg, South Africa; 6 Bristol Park Group of Hospitals, Nairobi, Kenya

**Keywords:** Post-abortion care, Abortion, Health systems, Capacity, Quality of care

## Abstract

**Background:**

In many parts of sub-Saharan Africa, access to abortion is legally restricted, which partly contributes to high incidence of unsafe abortion. This may result in unsafe abortion-related complications that demand long hospital stays, treatment and attendance by skilled health providers. There is however, limited knowledge on the capacity of public health facilities to deliver post-abortion care (PAC), and the spread of PAC services in these settings. We describe and discuss the preparedness and capacity of public health facilities to deliver complete and quality PAC services in Burkina Faso, Kenya and Nigeria.

**Methods:**

A cross-sectional survey of primary, secondary and tertiary-level public health facilities was conducted between November 2018 and February 2019 in the three countries. Data on signal functions (including information on essential equipment and supplies, staffing and training among others) for measuring the ability of health facilities to provide post-abortion services were collected and analyzed.

**Results:**

Across the three countries, fewer primary health facilities (ranging from 6.3–12.1% in Kenya and Burkina Faso) had the capacity to deliver on all components of basic PAC services. Approximately one-third (26–43%) of referral facilities across Burkina Faso, Kenya and Nigeria could provide comprehensive PAC services. Lack of trained staff, absence of necessary equipment and lack of PAC commodities and supplies were a main reason for inability to deliver specific PAC services (such as surgical procedures for abortion complications, blood transfusion and post-PAC contraceptive counselling). Further, the lack of capacity to refer acute PAC cases to higher-level facilities was identified as a key weakness in provision of post-abortion care services.

**Conclusions:**

Our findings reveal considerable gaps and weaknesses in the delivery of basic and comprehensive PAC within the three countries, linked to both the legal and policy contexts for abortion as well as broad health system challenges in the countries. There is a need for increased investments by governments to strengthen the capacity of primary, secondary and tertiary public health facilities to deliver quality PAC services, in order to increase access to PAC and avert preventable maternal mortalities.

**Supplementary Information:**

The online version contains supplementary material available at 10.1186/s12913-022-07873-y.

## Background

Currently, about 90% of women of childbearing age in Africa live in contexts with restrictive abortion laws [1], and the risk of dying from an unsafe abortion is highest in Africa [2]. Every year, between 4.7–13.2% of maternal deaths can be linked to unsafe abortion [3]. Recognizing this, at the 1994 International Conference on Population and Development (ICPD) in Cairo, 179 governments pledged to guarantee quality Post-Abortion Care (PAC) services. PAC is an integrated service delivery model that includes a set of maternal health and family planning interventions that are both curative and preventative [4]. In 2015, countries further adopted the Sustainable Development Goals (SDGs), aiming to reduce the global maternal mortality ratio (MMR) to less than 70 for every 100,000 live births [5]. Consequently, countries have developed national policies to improve provision of PAC as a public health necessity.

This paper uses a multi-country approach to assess the extent to which the health system is prepared to deliver PAC in three sub-Saharan Africa (SSA) countries (Burkina Faso, Kenya, and Nigeria). These countries possess varying national laws and policies on abortion and PAC [6], but show overall similarities in general state of health infrastructure [7]. For instance, abortion is legally restricted in all three countries [8–11]. Burkina Faso was among the first SSA countries to change its law from total prohibition to allowing abortion in preservation of a woman’s health [12]. Currently in Burkina Faso, abortion is legally permitted to save the life and protect the health of a pregnant woman, and in cases of rape, incest or severe fetal impairment [8]. In Kenya, abortion is not permitted unless, in the opinion of a trained health professional, there is need for emergency treatment, or the life or health of the mother is in danger, or if permitted by any other written law [11]. In 2012, the Ministry of Health launched the standards and guidelines for reducing maternal mortality due to abortion, which were briefly withdrawn and reinstated by the Kenyan high court creating some confusion around PAC provision including disruption of PAC training to providers, supplies and commodities to health facilities [13]. Nigeria presents a peculiar case where abortion legal frameworks vary by jurisdiction with about three legal systems applicable to abortion: the penal code applicable in the northern states, the criminal code in the southern states and across the other states; while Sharia penal legislation is applicable in 12 Northern states [10, 14]. In general, abortion is illegal unless done to save the life and health of the mother; specific states have extended conditions under which women can obtain abortion to include rape and incest [10]. As such, women of diverse social and demographic backgrounds within these countries, in need of safe termination of pregnancy resort to unsafe abortion methods and procedures, resulting in fatalities and a range of complications that require treatment, long hospital admissions, intensive care, and attendance by highly skilled, yet scarce healthcare personnel [15]. In Burkina Faso, about 105,000 abortions were induced in 2012 (an induced abortion rate of 25/1000 women aged 15–49), with a considerable proportion being unsafe [16]. In Kenya, about 500,000 induced abortions occurred in 2012 (rate of 48/1000 women), 75% of which presented with moderate to severe complications [17], while in Nigeria, about 1.25 million induced abortions occurred in 2012 (rate of 33 abortions/1000 women), and about 212,000 women were treated for complications of unsafe abortion [18]. A more recent study in 2018 showed that abortions are much more common in Nigeria (45.8 abortions per 1000 women) [19]. Despite post-abortion care being a public health imperative, a considerable proportion of women are unable to access quality PAC services in much of SSA [20]. In 2012, almost 285,000 women who had induced abortions in Nigeria experienced complications serious enough to require treatment, but could not receive the medical care they needed [18]. Similarly, 30% of women in Kenya and 40% in Burkina Faso did not receive the appropriate medical care following abortion-related complications [16, 17]. Several barriers impede timely access to PAC services including the varying degrees of legal restrictions on abortion that may constitute barriers to PAC [21], in addition to low capacity of health facilities to provide quality PAC services [22, 23] and stigma [24, 25].

There is broad consensus that maximizing access and utilization of PAC could reduce poor outcomes associated with abortion, even though expanding PAC alone is insufficient to avert abortion-related complications and deaths [4]. A complex interplay exists between quality care, PAC patient experiences and health outcomes. Even so, ensuring access to effective clinical and non-clinical PAC interventions, strengthening the health infrastructure (including for PAC signal functions), and having trained staff with optimum skills and a positive attitude could address abortion complications and prevent future unintended pregnancies. In Kenya for instance, the high incidence of repeat abortions among PAC clients raises questions about quality of post-abortion care available for women, especially PAC contraceptive counseling [25, 26]. Quality of healthcare is increasingly recognized as a core pillar of health systems reforms globally [27], with significant commitments toward strengthening health systems preparedness to address users’ needs and expectations. While abortion rates may not differ much by legality, abortion-related mortality rates differ significantly across settings. These variations in abortion-related mortality can be associated with inequitable access to safe abortion procedures as well as limited access to quality PAC services [28]. Thus, understanding the capability of health facilities to deliver quality and comprehensive PAC is critical in consolidating efforts towards reducing maternal mortality. So far, quality of PAC has been documented using a tripod framework for assessing healthcare quality that includes structural (facility infrastructure, management and staffing), process (technical quality and patient experience) and outcome (patient satisfaction, return visits and clinical outcomes) indicators [29]. Signal functions were initially designed by the United Nations in 1997 to monitor and improve provision of eight emergency obstetric care indicators [30]. However, over time, structural and process quality indicators for PAC have been assessed using signal function surveys in health facilities [23, 30, 31]. So far, several studies have pointed to a low capacity of health systems to provide PAC. Owolabi et al. (2019) conducted a multicountry analysis using signal functions in 10 countries to assess the health systems’ capacity to provide PAC [23]. They reported critical gaps in the provision of basic and comprehensive PAC across all facilities that offer delivery services. Such findings suggested weaknesses in meeting international commitments to address the consequences of unsafe abortion and the capacity of health systems to provide PAC. Similarly, Bell et al. (2021) described the PAC availability, readiness and accessibility of PAC services in Nigeria and Côte d’Ivoire. Only 48% of facilities in Nigeria could provide basic PAC services, with greater PAC capabilities in Côte d’Ivoire (70.5%) [28]. Another study by Owolabi et al. (2021) described the availability and capacity of health facilities to deliver PAC and SAC in Ghana, and noted that less than 20% of facilities at various levels could provide basic or comprehensive PAC, with least PAC capabilities in primary facilities and in rural areas [32]. In Zimbabwe, only 21% of facilities had basic PAC capability and 10% of referral facilities had comprehensive capability, and only one-fourth of PAC patients were treated with the appropriate medical procedure [31].

In this study, we examine the state of preparedness of public health facilities to deliver basic and comprehensive PAC in three SSA countries - Burkina Faso, Kenya and Nigeria, where safe abortions are rarely legally permitted. Our focus on public health facilities is driven by the fact that government policies and investment largely target the public sector and facilities. Private facilities are mainly business oriented and may set their own quality standards beyond what is stipulated in national PAC guidelines and other maternal health frameworks. We utilize signal function questionnaires that contain indicators (i.e. the availability of staff, staff training, key equipment and supplies, and ability to perform various reproductive health services) [33, 34], to describe PAC capacity. Some of the previous studies described above utilized secondary data sources including the Service Provision Assessment surveys [28] and the Performance Monitoring and Accountability 2020 (PMA2020) to examine capacity of primary and referral-level health facilities to deliver PAC services [23]. This paper reinforces existing literature on PAC, and provides new data in Burkina Faso and Kenya and additional data in Nigeria on the status of PAC services. This is a growing field within abortion literature in light of health systems performance and it is therefore important to sustain the momentum of generating robust evidence that informs the discourse on health systems improvement. We also describe the distribution of PAC services across facility levels and highlight the key gaps in PAC service provision that are amenable to improvements.

## Methods

### Study contexts

This was a multi-country study to assess the preparedness of public health facilities to deliver PAC services in Burkina Faso, Kenya and Nigeria. The three countries offer both similar and dissimilar contexts for investigating the quality of PAC. For instance, abortion is largely restricted across the three countries, and they all report high incidences of unsafe abortion [8, 10, 11]. These settings offer worthy contexts to examine the preparedness of their health facilities to provide PAC services.

### Study design and population

A cross-sectional survey was conducted among a representative sample of primary, secondary and tertiary health facilities in the aforementioned countries. Health systems across the three countries are organized according to hierarchical levels. Health facility levels are generally categorized as primary, secondary and tertiary-levels. Primary health facilities are the first point of contact for the majority of community members’ health needs, and include community facilities, dispensaries and clinics. In Kenya, primary-level facilities handle the Kenya Essential Package for Health (KEPH), which encompass activities related to health promotion, preventive care, and curative services. Secondary facilities are mainly sub-regional and regional and serve as referral facilities for the primary-level facilities. They undertake curative and rehabilitative care and address a limited extent of preventive care and health promotion. Tertiary facilities are mainly national referral and teaching hospitals. All health facilities capable of conducting normal deliveries were included in our sample frame. Data was collected in facilities over a 30-day period between November 2018 and February 2019.

### Sampling and recruitment

A two stage stratified sampling procedure was used in each country, that is, a) the highest sub-national administrative units (i.e. counties in Kenya, states in Nigeria and regions in Burkina Faso), and b) the levels of health facilities. The sub-national levels represented by “counties”, “states” and “regions”, denote the geopolitical zone below national and above district levels. At the first stage, in each country, a random sample of six regions, counties or states was drawn, and excluding the administrative unit hosting the national capital regions – i.e. Centre in Burkina, Nairobi in Kenya, and Abuja – Federal Capital Territory (FCT) in Nigeria. Thereafter, the capital regions were added to the regions purposely to make seven regions/counties/states in each country.

The selected administrative units included, Burkina Faso (seven regions from the 13: Boucle du Mouhoun, Cascades, Centre, Centre-Ouest, Centre-sud, Haut-Bassins, and Nord); Kenya (seven counties from 47: Garissa, Kajiado, Kiambu, Laikipia, Mandera, Migori, and Nairobi); Nigeria (seven states from 36: namely Anambra, Bauchi, Cross-River, Edo, Federal Capital Territory, Kano and Kogi.

At the second stage, the researchers obtained from government records updated master lists of all public health facilities in the different sub-national units. Burkina Faso and Nigeria’s list were updated up to July 2018 while Kenya was updated in February 2018. A requisite sample of facilities in each country was determined using a formula for known populations and known proportion estimates by: ∆ = *z√ ((p (1-p))/n*).

To solve for *n* we made it the subject: $\left(n={\left(\frac{z}{\Delta }\right)}^2p\left(1-p\right)\right)$, and assumed a confidence interval of 95%, with z as 1.96, and ∆ as 0.05. In all cases, the known estimate *p* represented the proportion of facilities capable of providing PAC contraceptive counseling, which was the lowest measure for quality of PAC in Kenya (19.4%) and Nigeria (16%) [22, 35]. Because we did not find any recent estimate in Burkina Faso, we used the 50% proxy in order to generate the maximum sample size possible. These calculations yielded the number of facilities required for each country, and upon accounting for a response rate of approximately 93%, the estimated sample size of facilities was determined as follows: 414 in Burkina Faso, 259 in Kenya, and 223 in Nigeria.

The total sample size of health facilities was allocated to each of the seven sub-national units in each country depending on the population of eligible facilities in a specific region/county or state. Therefore, health facilities were randomly selected within each sub-national level with all tertiary health facilities included and a sample of primary and secondary health facilities. Eligible facilities were those that could provide normal delivery services, were publicly owned (government owned) and operational at the time of survey. As such, we excluded some specialized facilities including mental and spinal hospitals as well as military and prison hospitals known not to offer services to the public. Our focus on public health facilities is because government investments in health services primarily go to these facilities. During the survey, some facilities were dropped and replaced with similar facilities within the same locality, due to insecurity and travel inaccessibility. In addition, sampled facilities that declined to participate in the study were replaced with similar facilities from the sampling frame, which had been identified a priori.

### Data collection

Trained field workers visited each eligible facility and administered the signal functions questionnaire which had been adopted from previous versions [36, 37]. The questionnaire was further refined to the contexts following extensive discussions with experienced obstetricians and gynecologists in each country. The questionnaires captured details on availability of key equipment, supplies and commodities, staffing and staff training, facility operation hours and ability to perform various sexual and reproductive health services (Supplementary file 1). Uniform tools were used across all countries. However, some aspects were adapted to fit in national standards (e.g. facilities categorizations). We asked the providers whether they were currently providing the listed services (Table 1). Whenever the provider indicated that a particular service was currently unavailable, the next sets of questions probed for the reasons why the service is not available. In response, the providers listed all possible reasons why the service was unavailable at that time. The tools were pre-tested to enhance conceptual clarity and logical flow. At large referral hospitals, respondents were the head of the obstetrics and gynecology department, or a key obstetrician gynecologist working in the facility. However, at lower level facilities, a nurse, a midwife or another health worker who was knowledgeable on PAC services provided in the facility was interviewed. The quantitative data were collected using tablets and hosted on the SurveyCTO platform. Completed and verified data were uploaded unto the APHRC cloud server for safe storage. Spot-checks were performed on 5% of the sample by the lead for each country.

**Table 1:** Description of signal functions for basic and comprehensive PAC services

*Signal functions required in order to both basic and comprehensive PAC services (Expected of both*	
1. Removal of retained products of conception^a^	
2. Administration of parenteral antibiotics^a^	
3. Administration of parenteral uterotonics^a^	
4. Administration of intravenous fluids^b^	
5. Provision of at least one modern, short acting family planning method at time of survey^b^	
*Signal functions for basic PAC (only in primary health facilities)*	
6. Availability of a vehicle with fuel to transport patients needing referral ^b^	
7. Availability of staff capable of undertaking normal deliveries (present/on duty or on call for 24 h/7 days a week	
*Signal functions for comprehensive PAC (only in referral facilities)*	
8. Administration of blood transfusion^a^	
9. Conducting major abdominal surgery such as laparotomy and Hysterectomy (proxied with provision of caesarean section)^a^	
10. Provision of at least one long-acting, reversible or permanent family planning method^b^	
11. Has staff capable of doing caesarean sections on duty or who are on call for 24 h per day, 7 days per week^c^	

Source: Owolabi et al., (2019) [23]

^a^Health facilities reported whether they were providing the service

^b^Health facilities indicated availability of drugs or equipment, and also indicated the validity or functionality of the given item

^c^This was premised on availability of staff capable of conducting caesarean sections (would also capable of doing normal deliveries)

### Data analysis

Using the Ministry of Health master list of health facilities in Burkina Faso, Kenya and Nigeria, and the sampling frame of public facilities, we constructed facility-levels weights accounting for the sample design and adjusting for stratification by regions/counties/states, and facility non-response, as well as applying a finite population correction. The statistical analysis was conducted in Stata version 15.0 [38]. We therefore use weighted data to describe the capacity of health facilities to deliver PAC services. We drew from the Owolabi et al. (2019) approach and constructed composite or aggregate indicators of signal functions to provide basic and comprehensive PAC using a signal functions approach [23]. By calculating the availability of specific health interventions that are key to PAC—i.e., the signal functions—we measure the capacity for, and quality of, PAC from a health systems perspective. We do this by summating or combining sets of indicators that constitute the two delineated levels of care - basic and comprehensive PAC, that roughly correspond to care that should be provided at both the primary level and at the referral level hospitals respectively (Table 1). A key departure from the Owolabi approach is that under basic PAC indicators, we excluded the ability to communicate with referral facilities. This was mainly because this variable was not captured in our data collection tool and was proxied with having an established referral pathway between different health facilities. We also explored another level of analysis, again adopted from the Owolabi paper [23], which included developing case scenarios by excluding some PAC signal functions to have a less restrictive criterion at various stages. At first, we analyzed all PAC signal functions for each facility levels. Secondly, we excluded the availability of staff capable of conducting normal deliveries, thirdly, we excluded - staff with delivery capabilities; having a referral capacity; availability of short and long-acting, or permanent family planning methods. At the fourth stage, we examined PAC capability by excluding the ability of a facility to conduct referrals (through having a vehicle fueled). “Capacity” or “preparedness” was conceptualized as the ability of health facilities to deliver services based on signal function indicators [37]. Proportions of facilities capable of delivering basic and comprehensive PAC were generated.

## Results

A total of 414 (Burkina Faso), 253 (Kenya) and 227 (Nigeria) health facilities participated in the survey. Health facilities included both primary, secondary and tertiary-level hospitals as illustrated in the Table 2.

**Table 2:** Distribution of sampled health facilities by Country^a^

	Primary-level facilities	Secondary-level facilities	Tertiary-level facilities	Total (Overall facility response rate)
Burkina Faso	354 (94.5%)	56 (5.2%)	4 (0.4%)	414 (100%)
Kenya	211 (94.3%)	39 (5.4%)	3 (0.3%)	253 (97.6%)
Nigeria	92 (95.5)	124 (4.3%)	11 (0.2%)	227 (100%)

Typical services offered by the various levels of facilities

**Primary-level facilities**: offer health promotion and preventive care, and various curative services including prenatal, delivery and antenatal services; **Secondary facilities**: undertake curative and rehabilitative activities, to a limited extent preventive/ health promotion, and are a referral point for primary facilities; **Tertiary facilities**: referral point for primary/secondary facilities, and offer wide range of specialized services including major surgeries

^a^ All data are weighted

### Capacity of health facilities to deliver post-abortion care services

#### Capacity of primary health facilities to deliver basic PAC services

Less than one in ten primary-level facilities in Kenya (6.3%) and Nigeria (8.6%) had capacity to deliver all elements of basic PAC services, which include-treatment of complications, family planning counselling and contraceptive services, ability to refer patients needing referral (through presence of vehicle with fuel), and staff capable of conducting normal deliveries. Burkina Faso had relatively more (12.1%) primary health facilities with basic PAC capabilities. When we excluded staff with capabilities to conduct normal deliveries, the proportion of primary facilities capable of basic PAC remained constant in Nigeria, and changed by about two-percent in Burkina Faso and Kenya (Table 3). Similarly, upon excluding - staff with delivery capabilities, referral capacity, and availability of various family planning methods, basic PAC capacity improved by 4.2% in Burkina Faso, and no significant change in Kenya and Nigeria. However, the greatest change in basic PAC was seen when we excluded the ability to conduct referrals for patients needing emergency care at a higher-level facility.

**Table 3:** Primary-level facilities capable of providing basic PAC services^a^

	Burkina F; *N* = 354	Kenya; *N* = 211	Nigeria; *N* = 92
	n (%)		
Basic PAC (all indicators)	43 (12.1)	9 (6.3)	7 (8.6)
Basic PAC (excluding staff with delivery capabilities)	49 (13.8)	14 (8.4)	7 (8.6)
Basic PAC (excluding - staff with delivery capabilities; referral capacity; availability of short and long-acting, or permanent family planning methods)	64 (18.0)	14 (8.4)	8 (9.3)
Basic PAC (excluding referral capabilities, i.e. no vehicle with fuel)	229 (64.7)	62 (26.2)	19 (20.5)

^a^ All data are weighted

While less number of primary facilities in Burkina Faso, Kenya and Nigeria had the capacity to offer all the basic PAC services, a considerable proportion of these primary facilities could offer specific basic PAC services, and this varied across countries. For instance, a majority of primary-level facilities in Burkina Faso and Kenya (≥92%) could administer parenteral antibiotics and intravenous fluids compared to Nigeria (88.4%) (Table 4). The least capability score is noted on availability of transport for referral to higher-level facilities; approximately one-tenth (12.8%) of primary facilities in Kenya and Nigeria (11.8%) have vehicles/ambulances and one-fifth of primary-level facilities in Burkina Faso (21.1%). One service for which the primary facilities in Kenya fared rather poorly (below 50%) compared to Burkina Faso and Nigeria is the availability of staff to perform normal deliveries on normal duties or on call 24 h daily.

**Table 4:** Capability to provide basic post-abortion care signal functions among primary-level facilities^a^

	Burkina Faso *N* = 354(%)	Kenya *N* = 211 (%)	Nigeria *N* = 92 (%)
Remove retained products of conception*	90.7	64.7	78.1
Administer parenteral antibiotics*	99.4	93.3	88.4
Administer parenteral uterotonics*	98.6	58.6	81.4
Administer intravenous fluids†	98.9	92	89.2
Has vehicle with fuel to transport patients needing referral†	21.1	12.8	11.8
Has staff capable of undertaking normal deliveries on duty or who are on call for 24 h everyday	93.8	33.7	73.9
Provide at least one modern, short-acting family planning method at time of survey†	72.6	91.0	70.5

*Assessed on the basis of facility reporting if they had ever provided the service; †assessed on the basis of the availability and validity or functionality of a given item (drug or equipment) at the time of survey

^a^ All data are weighted

### Capacity of referral health facilities to deliver comprehensive PAC services

Across the three countries, just one-third of referral-level health facilities in Burkina Faso (30%) and Nigeria (25.8%) could deliver the entire package of comprehensive PAC services, compared to 42.9% in Kenya. These services included - treatment of complications, family planning counseling and contraceptive services, ability to conduct blood transfusion, major abdominal surgery, and having a vehicle with fuel for possible referral. After applying a less restrictive criterion to assess capacity of referral facilities to deliver comprehensive PAC, only Nigeria had notable improvements in the proportion of facilities capable of comprehensive PAC (Table 5).

**Table 5:** Referral-level facilities capable of providing comprehensive PAC services^a^

	Burkina F; *N* = 60	Kenya; *N* = 42	Nigeria; *N* = 135
	n (%)		
Comprehensive PAC (all indicators)	18 (30.0)	14 (42.9)	40 (25.8)
Comprehensive PAC (excluding staff with caesarean section ability working daily)	18 (30.0)	14 (42.9)	45 (29.9)
Comprehensive PAC (excluding - staff with caesarean abilities, availability of short and long-acting, or permanent family planning methods)	20 (33.3)	14 (42.9)	47 (32.1)
Comprehensive PAC (excluding referral capabilities, i.e. no vehicle with fuel)	20 (33.3)	16 (45.2)	62 (41.0)

^a^ All data are weighted

All tertiary level facilities reported high capacity for specific comprehensive PAC services. However, not all tertiary facilities in Nigeria had capabilities to facilitate PAC referrals, as was few facilities in Burkina Faso that could not undertake major abdominal surgery (Table 6). Ordinarily, this should not be expected of tertiary facilities, however we know that in many developing countries, some health facilities designated as tertiary could lack certain expected resources/infrastructure (e.g. ICU). There were differences between secondary and tertiary facilities and across the three countries. For instance, fewer secondary-level facilities in Burkina Faso (37.5%) had capacity to undertake a major abdominal surgery, such as laparotomy or hysterectomy, compared to Kenya (43.3%) and Nigeria (48.2%). Notably, only half (48.2%) of secondary facilities in Burkina Faso administer blood transfusion, compared to 61% in Kenya and 85.5% in Nigeria. A greater proportion of secondary facilities in Burkina Faso (80.4%) and Kenya 89.4% had vehicles for referral purposes, compared to 63.9% in Nigeria.

**Table 6:** Capability to provide comprehensive PAC signal functions among secondary and tertiary facilities^a^

	Burkina F (%)		Kenya (%)		Nigeria (%)	
	Secondary (*N* = 56)	Tertiary (*N* = 4)	Secondary (*N* = 38)	Tertiary (*N* = 4)	Secondary (*N* = 124)	Tertiary (*N* = 11)
Remove retained products of conception*	98.2	100	100	100	91.1	100
Administer parenteral antibiotics*	100	100	100	100	95.2	100
Administer parenteral uterotonics*	100	100	90.9	100	90.4	100
Administer intravenous fluids†	100	100	100	100	97.4	100
Has vehicle with fuel to conduct referral of patients needing †‡	80.4	100	89.4	100	63.9	88.9
Provide modern, short and long acting family planning method†	83.9	100	87.9	100	79.7	100
Administer a blood transfusion*	48.2	100	61.0	100	85.3	100
Undertake major abdominal surgery (laparotomy/hysterectomy)*	37.5	75	43.3	100	48.2	100
Has staff capable of doing caesarean sections daily	96.4	100	97.4	100	79.8	100

Key: *Assessed on the basis of facility reporting if they had ever provided the service; ^†^assessed on the basis of the availability and/or functionality of a given item (drug/equipment) at time of survey; ^‡^we assumed that staff who were capable of doing caesarean sections were also capable of doing normal deliveries, and therefore did not need to include this factors in comprehensive capability for PAC

^a^ All data are weighted

### Facility operation hours

Majority of facilities in Burkina Faso (98.3%) and in Nigeria (69.5%) operated every day for 24 h, while less than 40% of health facilities in Kenya did so. There were stark contrasts on the days in which facilities deliver contraceptives, for instance; only about 3% of all facilities in Kenya offered contraceptive services 24 h daily (including weekends), while over 94% in Burkina Faso do so, and 53% in Nigeria. Majority of facilities in Kenya (89.9%) nevertheless operate for 5 days for less than 24 h as seen in Table 7.

**Table 7:** Facility operation periods and provision of contraception products^a^

	Burkina Faso (%)	Kenya (%)	Nigeria (%)
**Operational days and time**			
7 days for 24 h/per day	98.3	33.0	69.5
5 days and less than 24 h/per day	0	56.1	18.1
Others	1.7	10.8	12.4
**Days and time when Contraceptive services are provided**			
7 days for 24 h/per day	95.5	2.6	53.1
5 days and less than 24 h/per day	0	89.9	14.8
Others	4.6	7.6	32.0

^a^ All data are weighted

### Reasons why some facilities could not deliver basic or comprehensive PAC services

**Fig. 1:**
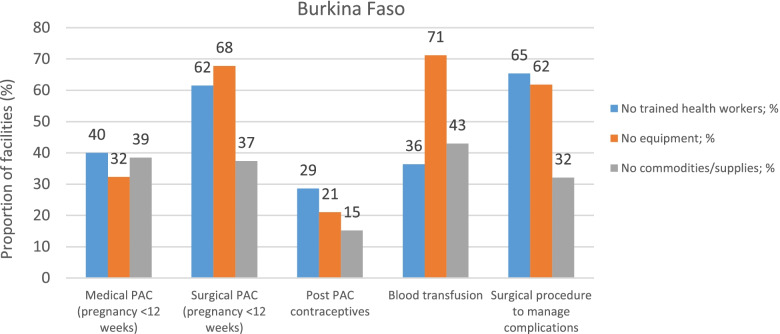
Reasons for not delivering PAC services in Burkina Faso

**Fig. 2:**
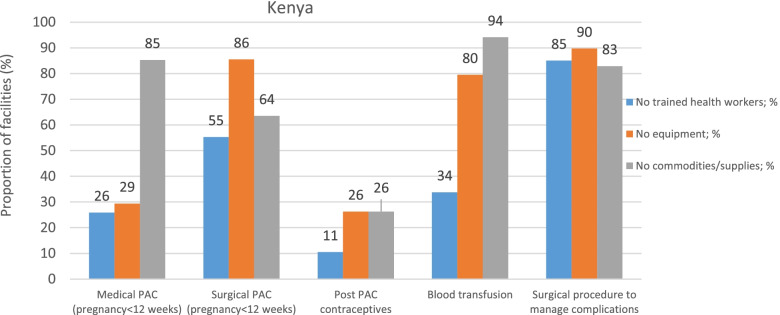
Reasons for not delivering PAC services in Kenya

**Fig. 3:**
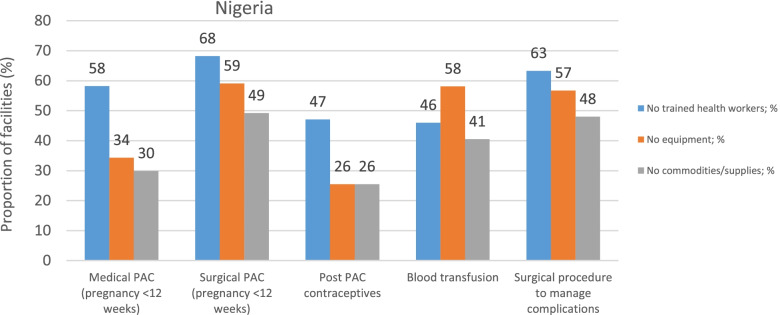
Reasons for not delivering PAC services in Nigeria

Respondents from Burkina Faso, Kenya and Nigeria, cited a number of reasons for the limited capacity to deliver basic and or comprehensive PAC services. Lack of skills and specific training on PAC services, lack of equipment, and unavailability of certain commodities and supplies for PAC, were commonplace across Burkina Faso (Fig. 1), Kenya (Fig. 2) and Nigeria (Fig. 3). In Burkina Faso and Kenya, the greatest impediment to delivery of surgical procedures that manage abortion complications including surgical PAC for pregnancies below 12 weeks of gestations was the lack of trained staff and absence of the necessary equipment. While medical evacuation procedures were largely hampered by the lack of trained providers in Burkina Faso and Nigeria, in Kenya the stock-outs of supplies and commodities were main factors. Inability to deliver blood transfusions was mainly attributed to the lack of supplies and commodities in Kenya, as opposed to the lack of equipment in Burkina Faso and Nigeria.

## Discussion

Across the three countries, data on availability and distribution of PAC services showed the poor state of healthcare for women who present at public health facilities with abortion-related complications. Overall, very few primary health facilities in the three countries (i.e. Kenya- 6.3%, Nigeria-8.6%, and Burkina Faso-12.1%) had capacity to deliver on all basic PAC services. Notably, Burkina Faso had twice and three-times a greater capacity for basic PAC compared to Kenya and Nigeria respectively. These findings are consistent with previous studies that reported low capacity of health systems for PAC services. For instance, in Zambia, only 2.6% of facilities could deliver basic PAC services [34]. In addition, other studies have also reported lower figures for basic PAC in Tanzania, Uganda, Rwanda and Namibia [36, 37, 39]. On the contrary, other recent studies in countries such as Malawi (29%) and Senegal (16%) had significantly higher proportions of facilities able to deliver basic PAC services [40]. Higher basic PAC capacity in Burkina Faso compared to Kenya and Nigeria, may reflect differences in groupings of primary facilities and their capabilities, but also the national government’s prioritization and investments in general maternal health services across various levels of health facilities, specifically, quality post-abortion care services. Approximately one-third (30–43%) referral facilities in the focus countries were capable of providing all elements of comprehensive PAC services. Referral-level facilities in Kenya had greater comprehensive PAC capabilities compared to similar facilities in Burkina Faso and Nigeria. The proportions reported in this study are higher compared to those in a Zambian study (0.3%). Such disparities could be due to the higher threshold (12 indicators) used in Zambian work, which did not create categorization for the clusters of services potentially available in primary facilities, but rather examined all facilities as similar. Nevertheless, results from surveys on comprehensive PAC services in Uganda, Senegal, Rwanda, Namibia and Kenya were largely similar to our findings [23]. Importantly, the existing PAC capacities at referral facilities are still regarded as low and need urgent prioritization. Referral facilities are the endpoint of critical and specialized care to PAC patients, and such services can be urgent without which women may experience permanent disabilities and eventual death. The study findings are thus very concerning much more for Kenya and Burkina Faso, where even after applying less-stricter measures of comprehensive PAC (excluding staff availability and post-abortion family planning services to at least one long-acting or short-acting method), there were no significant changes in their overall PAC capacities. There is consensus that capacity to deliver post abortion interventions must entail a combination of personal skills of health staff and health facility resources including supplies and commodities such as blood for transfusion and drugs such as Misoprostol. As such, gaps and weaknesses in either result in poor care experiences, care costs and health outcomes. Health providers training is key, allowing them to assess the clinical condition of PAC patients, diagnose, and prescribe appropriate treatment for the patient. There are opportunities to enhance such training through pre-service and periodic in-service training. The absence of training means providers mostly act as a relaying belt by referring all patients that come their way – further increasing delays to care and cost of care.

While the abortion laws do not differ much across the three countries, the national laws are generally restrictive, and overlap with other policy and cultural restrictions to limit availability and access to PAC [20]. For instance in Kenya, inconsistencies and confusion around abortion law, and the Ministry of Health move to withdraw the standards and guidelines for reducing abortion related mortality in 2013, led to fear, confusion, and disrupted both PAC provider training and supply of commodities and equipment. Pervasive religious influences in Nigeria discourages women from presenting for PAC, and health providers from offering PAC including contraceptive guidance and services. In Burkina Faso, structural weaknesses in PAC services are more linked to health system weaknesses, translating into supply chain issues, and the lack of equipment and commodities.

By using signal functions indicators to examine preparedness of facilities to deliver specific basic PAC services, this study was able to highlight critical strengths and challenges in delivering basic PAC at the primary level, and also offer an opportunity to compare across countries. In general, no country had all primary level facilities capable of providing all basic PAC services, with specific gaps in the availability of vehicles with fuel (preferably ambulance) to facilitate seamless transfer of patients to the proximate higher facility. Even though lower level referral facilities (especially secondary facilities) were able to deliver many of the PAC signal functions, they were unable to provide two of the most essential interventions to manage life threatening abortion complications, blood transfusions and abdominal surgeries. These interventions are the cornerstone of comprehensive PAC clinically and in their absence then it is almost not worth sending women to these facilities. We know that primary facilities are more prevalent in these contexts, which implies that they are often the first point of contact for medical emergencies including those arising from unsafe abortions [20, 28]. Improved preparedness of these facilities to provide PAC services is fundamental to saving the lives of women and girls.

In context, our findings suggest that many women may not be able to receive appropriate PAC at the nearest health facilities (normally primary facilities) in these countries. For women who access referral-level facilities for PAC, either through referral from primary care or through bypassing primary care altogether, there is no guarantee that they will be successful at getting needed care to manage abortion-related complications. These limitations in access to basic or comprehensive PAC may result in abortion related mortalities. The weak referral capacities at primary facilities means that women may have to facilitate their own transfer when they require critical care. In rural and remote settings where transportation is poor, women may be subjected to tortuous journeys moving from one facility to the other. Relative variability exists between countries in the provision of specific PAC services, reflective of the political, legal and policy environment, distinct health system structures and expectations of each level of facility, regardless of our broad classification into primary, secondary and referral levels for analysis.

### Strengths and limitation

Among the key strengths of this study are that we collected primary data on the health system indicators of PAC from a cross-section of health facilities in Burkina Faso, Kenya and Nigeria, and complemented this survey data with in-depth interviews from key providers of PAC and related services. This is an improvement from previous studies that utilized service provision assessment data, which are often not collected to measure PAC services but maternal health generally. Further, we attempt to measure the quality of PAC at different levels of health service delivery across the three countries and report on critical gaps in the provision of PAC at all facilities that offer delivery services. Nonetheless, there are certain limitations to the study. First, while the study assesses the structural and process indicators [41, 42], it fails to examine patient clinical outcomes as part of the healthcare quality framework. This implies that we are unable to link facility-level structural preparedness to deliver PAC to specific clinical outcomes of patients seeking PAC services. We however collected data on the process indicators and conducted in-depth interviews with patients to understand their experiences and satisfaction levels with the PAC services they received. Future studies should attempt to collect both structural, process and care outcome data to better draw conclusions on PAC service delivery. Considering there are five essential components of comprehensive PAC, this study address three - treatment of abortion complications, contraceptive and family planning services, and the provision of reproductive and other health services. Because of limited resources, this survey did not assess counseling to identify and respond to women’s emotional and physical needs including stigma, fear of prosecution and service provider partnerships for prevention, mobilization of resources and ensuring that health services reflect and meet community expectations and needs. This means that while the analysis presented in this paper is extensive, it does not cover the other elements of comprehensive extent of PAC service provision. We however conducted qualitative in-depth interviews, which will provide insightful details on the other components of comprehensive PAC. We recommend that future studies directly examine PAC practices on psychological counselling and how community and health provider linkages are implemented to leverage community resources that enhance uptake of contraceptives, prevent unsafe abortion and promote utilization of PAC services. Finally, by not including private facilities in our sample, our findings do not fully reflect the status of PAC in the study countries. However, we were keen on public sector health facilities because our intention was to influence public policy, which managed by the government, and where majority women visit for PAC.

## Conclusions

In summary, the study found low capacity of primary and referral facilities to provide PAC, and very few primary or secondary facilities could deliver all elements of basic and comprehensive PAC respectively. While PAC is a critical emergency service, results from this study point to severe gaps and weaknesses in delivery of PAC in Burkina Faso, Kenya and Nigeria, which may reflect the variations in political, legal and policy environment in the countries. To achieve improved maternal mortality targets, there is need for governments to increase investments and build infrastructure that strengthens the capacity of health facilities to deliver quality PAC services including training of health providers, supplies and commodities, and referral to higher-level facilities.

## Supplementary Information

**Additional file 1.** Supplementary file 1.

## Data Availability

Data are available from the authors.

## Abbreviations

APHRC: African Population and Health Research Center
FCT: Federal Capital Territory
ICPD: International Conference on Population and Development
KEPH: Kenya Essential Package for Health
KNH: Kenyatta National Hospital
MMR: Maternal Mortality Ratio
MoH: Ministry of Health
NACOSTI: National Commission for Science, Technology & Innovation
PAC: Post-Abortion Care
PMA2020: Performance Monitoring and Accountability 2020
SDGs: Sustainable Development Goals
SSA: sub-Saharan Africa
