# Supplementary material for: Health systems’ preparedness to provide post-abortion care: assessment of health facilities in Burkina Faso, Kenya and Nigeria
Source: BMC Health Serv Res. 2022 Apr 22;22:536. doi: 10.1186/s12913-022-07873-y (PMC9027923; doi:10.1186/s12913-022-07873-y)
Supplement: Supplementary file 1 — Additional file 1. Supplementary file 1. [file 12913_2022_7873_MOESM1_ESM.pdf]

Supplimentary File 1

Signal Functions Assessment Tool

Interviewer information

Date of Data collection								Name of Investigator	Investigator ID		Time interview started							
D	D	/	M	M	/	Y	Y				H	H	:	M	M	A/P	M	

1. Name of Facility	2. Level of facility	3. Ward
4. County/State/Region		5. GPS coordinates (For in-person interviews)

Type of health facility	Tick one	Operating agency	Tick one
A. Tertiary hospital (Level 3)	☐	1. Government	☐
B. Provincial hospital (Level 2)	☐	2. Privately owned (CIRCLE TYPE: 1. NGO 2. Religious body 3.private for profit 4. Industrial (Mine hospitals)	☐
C. District hospital (Level 1)	☐		
D. Health centre	☐	3. Other (please indicate) ______________________________	☐
E. Health post	☐	4. Other (please indicate) ______________________________	☐
F. Other (please indicate) ______________________________	☐		

STAFF available at facility (In LEVEL 3 hospitals fill this in for the gynaecologic/obstetric emergency ward where patients are managed. FOR all other fill it in for the whole hospital)		Work hours	Circle the appropriate answer	
How many obstetricians are registered to work at this facility normally?	☐☐	Is the facility open on:		
How many obstetricians are present at this facility right now?	☐☐	Monday (Circle the appropriate answer)	Yes	No
How many obstetricians are on call overnight everyday?	☐☐	Tuesday (Circle the appropriate answer)	Yes	No
		Wednesday (Circle the appropriate answer)	Yes	No
		Thursday (Circle the appropriate answer)	Yes	No
		Friday (Circle the appropriate answer)	Yes	No
		Saturday (Circle the appropriate answer)	Yes	No
		Sunday (Circle the appropriate answer)	Yes	No
Is there an obstetrician present for 24 hours everyday?	Yes / No	Is it open for 24 hours on:		
How many other doctors (MBBS) are registered to work at this facility normally?	☐☐	Monday (Circle the appropriate answer)	Yes	No
How many other doctors (MBBS) are present at this facility right now?	☐☐	Tuesday (Circle the appropriate answer)	Yes	No
		Wednesday (Circle the appropriate answer)	Yes	No
		Thursday (Circle the appropriate answer)	Yes	No
		Friday (Circle the appropriate answer)	Yes	No

How many other doctors (MBBS) are on call overnight everyday?

Are there any other doctors (MBBS) present for 24 hours everyday? Yes No

How many clinical officers are present at this facility right now?

How many clinical officers are on call overnight everyday? Yes No

Is there a clinical officer present for 24 hours everyday?

How many midwives/nurses are registered to work at this facility normally?

How many midwives/nurses are present at this facility right now?

How many midwives/nurses are on call overnight everyday?

Is there a midwife/nurse present for 24 hours everyday? Yes No

How many anaesthetists are registered to work at this facility normally?

How many anaesthetists are present at this facility right now?

How many anaesthetists are on call overnight everyday?

Is there an anaesthetists present for 24 hours everyday? Yes No

Is there any other cadre of health professional with comprehensive abortion care training registered to work at this facility normally? Yes No

What are the other cadres of health professionals who provide comprehnsive abortion care at this facility?

1._______________

2.________________

3.__________________

How many other cadre of health professional with comprehensive abortion care
[illegible]

Saturday (Circle the appropriate answer) Yes No

Sunday (Circle the appropriate answer) Yes No

Is family planning provided on:

Monday (Circle the appropriate answer) Yes No

Tuesday (Circle the appropriate answer) Yes No

Wednesday (Circle the appropriate answer) Yes No

Thursday (Circle the appropriate answer) Yes No

Friday (Circle the appropriate answer) Yes No

Saturday (Circle the appropriate answer) Yes No

Sunday (Circle the appropriate answer) Yes No

Is family planning provided for 24 hours on:

Monday (Circle the appropriate answer) Yes No

Tuesday (Circle the appropriate answer) Yes No

Wednesday (Circle the appropriate answer) Yes No

Thursday (Circle the appropriate answer) Yes No

Friday (Circle the appropriate answer) Yes No

Saturday (Circle the appropriate answer) Yes No

Sunday (Circle the appropriate answer) Yes No

training are registered to work at this facility normally?

How many other cadre of health professional with comprehensive abortion care training are present at this facility right now? [|]

How many other cadre of health professional with comprehensive abortion care training are on call overnight everyday? [|]

Is there any other cadre of health professional with comprehensive abortion care training present for 24 hours everyday? [|]

Name of respondent to this section: ____________________ Position of respondent to this section in facility ____________________

Instructions

Answer the following questions about signal functions in this facility related to emergency obstetric care including abortion/post abortion care by interviewing the health workers in the gynaecology ward/Emergency room/family planning clinic and other departments; reviewing facility registers; and observation (as much as possible).

Record whether the function has been carried out in the last 3 months. If not, indicate why it has not been performed using the categories given below. Then record whether it has been done in the past 12 months.

Categories of answers for non-performance of a function

A.		No health workers available
	1	Health workers required for this function are not sufficient or available at all
B.		No trained health workers
	1	The authorized staff are available but untrained
	2	They are trained but not confident enough to perform the function
C.		No equipment (e.g. machines/beds)
	1	Large equipment are not available e.g. machines, beds
D.		No commodities/supplies
	1	Drugs/family planning commodities needed are not available
E.		Against hospital/management policies
	2	The procedure is against managements policies
	3	Staff are encouraged to do other procedures for some reason
	4	Lack of supervision
F.		No cases requiring this procedure
	1	No client needing this function has come to the facility in this period (Not necessary).
G.		Against the workers morals/ethics
	2	Staff are morally or ethically uncomfortable with the procedure

SECTION 1		
Q.	A. Can your facility perform this procedure?	
1	**Pregnancy testing**	**YES**……………….skip to 1B<br><br>**NO**<br>**WHY not?**<br>***(circle all answers indicated)*** …….**then skip to 2A**<br>A. No health workers available<br>B. No trained health workers<br>C. No equipment e.g. machines or beds<br>D. No commodities or supplies<br>E. Against hospital or management policies<br>F. No cases requiring the procedure<br>G. Against the workers morals/ethics
2	**Parenteral administration of antibiotics to women**	**YES……………….skip to 2B**<br><br>**NO**<br>**WHY not?**<br>***(circle all answers indicated)* …….then skip to 3A**<br>1. No health workers available<br>2. No trained health workers<br>3. No equipment e.g. machines or beds<br>4. No commodities or supplies<br>5. Against hospital or management policies<br>6. No cases requiring the procedure<br>7. Against the workers morals/ethics
3	Administration of uterotonic oxytocics	**YES**……………….skip to 3B<br><br>**NO**<br>**WHY not?**<br>***(circle all answers indicated)*** ………. **then Skip to 4A**<br>A. No health workers available<br>B. No trained health workers<br>C. No equipment e.g. machines or beds<br>D. No commodities or supplies<br>E. Against hospital or management policies

		F. No cases requiring the procedure<br>G. Against the workers morals/ethics
4	Administration of IV fluids to woman	**YES**………………. 4B<br><br>**NO**<br>**WHY not?**<br>***(circle all answers indicated)*** ……….**then Skip to 5A**<br>A. No health workers available<br>B. No trained health workers<br>C. No equipment e.g. machines or beds<br>D. No commodities or supplies<br>E. Against hospital or management policies<br>F. No cases requiring the procedure<br>G. Against the workers morals/ethics
5	Administer parenteral anticonvulsants	**YES**………………. 5B<br><br>**NO**<br>**WHY not?**<br>***(circle all answers indicated)*** ……….**then Skip to 6A**<br>1. No health workers available<br>2. No trained health workers<br>3. No equipment e.g. machines or beds<br>4. No commodities or supplies<br>5. Against hospital or management policies<br>6. No cases requiring the procedure<br>**7.** Against the workers morals/ethics
6	Use partographs to monitor and manage labour	**YES**………………. 6B<br><br>**NO**<br>**WHY not?**<br>***(circle all answers indicated)*** ……….**then Skip to 7A**<br>A. No health workers available<br>B. No trained health workers<br>C. No equipment e.g. machines or beds<br>D. No commodities or supplies<br>E. Against hospital or management policies<br>F. No cases requiring the procedure<br>G. Against the workers morals/ethics
7	Perform active management of third stage of labour routine for all deliveries	**YES**………………. 8A<br><br>**NO**<br>**WHY not?**<br>***(circle all answers indicated)*** ……….**then Skip to 8A**<br>A. No health workers available<br>B. No trained health workers

		C. No equipment e.g. machines or beds<br>D. No commodities or supplies<br>E. Against hospital or management policies<br>F. No cases requiring the procedure<br>G. Against the workers morals/ethics
8	Perform manual removal of placenta	**YES**……………… 8B<br><br>**NO**<br>**WHY not?**<br>***(circle all answers indicated)*** ……….**then Skip to 4A**<br>A. No health workers available<br>B. No trained health workers<br>C. No equipment e.g. machines or beds<br>D. No commodities or supplies<br>E. Against hospital or management policies<br>F. No cases requiring the procedure<br>G. Against the workers morals/ethics
9	Medical post abortion care (PAC). Removal of retained products of conception for uterine size less than12 weeks or first trimester pregnancies by medications (PAC)	**YES**………………. 9B<br><br>**What type of medications do you use?**<br>Mifepristone and misoprostol combination (medabon)…………………………………………..…..1<br>Misoprostol (cytotec) alone..........................2<br>Other (specify)________________________3<br>Can you remember brand name??______________________________
		**NO**<br>**WHY not?**<br>***(circle all answers indicated)*** ……….**then skip to 5A**<br>1. No health workers available<br>2. No trained health workers<br>3. No equipment e.g. machines or beds<br>4. No commodities or supplies<br>5. Against hospital or management policies<br>6. No cases requiring the procedure<br>**7.** Against the workers morals/ethics
10		**YES**……………… 10B<br><br>**What procedures do you use?**<br>Vacuum aspiration (VA)……………..1 Dilatation and Curettage...............................2<br>Other (specify)________________________3

	Surgical post abortion care (PAC). Removal of retained products of conception for uterine size less than 12 weeks or first trimester pregnancies surgically	
		**NO**<br>**WHY not?**<br>***(circle all answers indicated)*** ……….**then skip to 6A**<br>1. No health workers available<br>2. No trained health workers<br>3. No equipment e.g. machines or beds<br>4. No commodities or supplies<br>5. Against hospital or management policies<br>6. No cases requiring the procedure<br>**7.** Against the workers morals/ethics
11	Medical voluntary termination of pregnancy (TOP) for uterine size less than 12 weeks or first trimester pregnancies by medications	**YES**……………… 11B<br><br>**What type of medications do you use?**<br>Mifepristone and misoprostol combination..1 Misoprostol alone..2<br>Other (specify)________________________3<br>Can you remember brand name??______________________________
		**NO**<br>**WHY not?**<br>***(circle all answers indicated)*** ……….**then Skip to 7A**<br>1. No health workers available<br>2. No trained health workers<br>3. No equipment e.g. machines or beds<br>4. No commodities or supplies<br>5. Against hospital or management policies<br>6. No cases requiring the procedure<br>**7.** Against the workers morals/ethics
12	Surgical voluntary termination of pregnancy (TOP) for uterine size less than12 weeks or first trimester pregnancies?	**YES**……………… 12B<br><br>**What procedures do you use?**<br>Vacuum aspiration (VA)……………..1 Dilatation and Curettage...............................2<br>Other (specify)________________________3

	pregnancies?	**NO**<br>**WHY not?**<br>***(circle all answers indicated)*** ………**then Skip to 8A**<br>1. No health workers available<br>2. No trained health workers<br>3. No equipment e.g. machines or beds<br>4. No commodities or supplies<br>5. Against hospital or management policies<br>6. No cases requiring the procedure<br>**7.** Against the workers morals/ethics
13	Provision of contraceptives after PAC or voluntary termination of pregnancy (TOP)	**YES**……………… 13B<br><br>**What contraceptives does this facility provide/prescribe?**<br>***(Circle all that apply)***<br>**01.** Combined oral contraceptive pills (e.g. oralcon)<br>**02.** Progestin-only contraceptive pills (e.g. microlut)<br>**03.** Combined injectable contraceptives<br>**04.** Progestin-only injectable contraceptives (e.g. Depo)<br>**05.** Male condoms<br>**06.** Female condoms<br>**07.** Intrauterine contraceptive device (IUCD)<br>**08.** Implant<br>**09.** Emergency contraceptive pills<br>**10.** Male sterilization<br>**11.** Female sterilization
		**NO**<br>**WHY not?**<br>***(circle all answers indicated)*** ……….**then Skip to 9A**<br>A. No health workers available<br>B. No trained health workers<br>C. No equipment e.g. machines or beds<br>D. No commodities or supplies<br>E. Against hospital or management policies<br>F. No cases requiring the procedure<br>G. Against the workers morals/ethics
14	**Does family planning provision take place in the same place where they have PAC or TOP or not**	**1. Same room where they have PAC/TOP with the same provider**<br>**2. Same room where they have PAC/TOP with a different provider**<br>**3. Another place within the health facility compound with the same provider**<br>**4. Another place within the health facility compound with a different provider**<br>**5. Another place outside this health facility**
15		**YES**………………. 14B<br><br>**What type of medications do you use?**<br>Mifepristone and misoprostol combination (medabon)…………………………………………..…..1<br>Misoprostol (cytotec) alone..........................2<br>Other (specify)________________________3

	Medical post abortion care (PAC). Removal of retained products of conception for uterine size more than (or equal to)greater than12 weeks (second trimester)	Can you remember brand name??______________________________
		**NO**<br>**WHY not?**<br>***(circle all answers indicated)*** **?**…….**then Skip to 11A**<br>A. No health workers available<br>B. No trained health workers<br>C. No equipment e.g. machines or beds<br>D. No commodities or supplies<br>E. Against hospital or management policies<br>F. No cases requiring the procedure<br>G. Against the workers morals/ethics
16	Surgical post abortion care (PAC). Removal of retained products of conception for uterine size greater than12 weeks (second trimester)	**YES**………………. 15B<br><br>**What procedures do you use?**<br>Vacuum aspiration (VA)……………..1 Dilatation and Curettage...............................2<br>Other (specify)________________________3
		**NO**<br>**WHY not?**<br>***(circle all answers indicated)*** …….**then Skip to 12A**<br>1. No health workers available<br>2. No trained health workers<br>3. No equipment e.g. machines or beds<br>4. No commodities or supplies<br>5. Against hospital or management policies<br>6. No cases requiring the procedure<br>7. Against the workers morals/ethics
17	Provision of voluntary termination of	**YES**………………. 16B<br><br>**What type of medications do you use?**<br>Mifepristone and misoprostol combination (medabon)…………………………………………..…..1<br>Misoprostol (cytotec) alone..........................2<br>Other (specify)________________________3<br>Can you remember brand name??______________________________

	pregnancy for uterine size greater than12 weeks(second trimester) by medications	
		**NO**<br>**WHY not?**<br>***(circle all answers indicated)*** ……….**then skip to 13C**<br>A. No health workers available<br>B. No trained health workers<br>C. No equipment e.g. machines or beds<br>D. No commodities or supplies<br>E. Against hospital or management policies<br>F. No cases requiring the procedure<br>G. Against the workers morals/ethics
18	Provision of voluntary termination of pregnancy for uterine size greater than 12 weeks(second trimester) surgically	**YES**………………. 17B<br>**What procedures do you use?**<br><br>Vacuum aspiration (VA)……………..1 Dilatation and Curettage..............................2<br>Dilatation and evacuation…………………………3<br>Other (specify)________________________4
		**NO**<br>**WHY not?**<br>***(circle all answers indicated)*** …….**then skip to 14A**<br>A. No health workers available<br>B. No trained health workers<br>C. No equipment e.g. machines or beds<br>D. No commodities or supplies<br>E. Against hospital or management policies<br>F. No cases requiring the procedure<br>**G.** Against the workers morals/ethics
19	Encourage Immediate and exclusive breastfeeding to all women	**YES**………………. 19<br><br>**NO**<br>**WHY not?**<br>***(circle all answers indicated)*** ……….**then Skip to 20 A**<br>A. No health workers available<br>B. No trained health workers<br>C. No equipment e.g. machines or beds<br>D. No commodities or supplies<br>E. Against hospital or management policies<br>F. No cases requiring the procedure<br>G. Against the workers morals/ethics
20		**YES**………………. 20

	Hygienic cord care (cut with sterile item and apply disinfectant to tip and stump, and no application of other substances) for all	**NO**<br>**WHY not?**<br>***(circle all answers indicated)*** ……….**then Skip to 21A**<br>A. No health workers available<br>B. No trained health workers<br>C. No equipment e.g. machines or beds<br>D. No commodities or supplies<br>E. Against hospital or management policies<br>F. No cases requiring the procedure<br>G. Against the workers morals/ethics
21	Thermal protection (drying baby immediately after birth and wrapping) for all (or say routinely)	**YES**………………. 21<br><br>**NO**<br>**WHY not?**<br>***(circle all answers indicated)*** ……….**then Skip to 22A**<br>A. No health workers available<br>B. No trained health workers<br>C. No equipment e.g. machines or beds<br>D. No commodities or supplies<br>E. Against hospital or management policies<br>F. No cases requiring the procedure<br>G. Against the workers morals/ethics
22	Administer IV fluids to baby	**YES**………………. 21B<br><br>**NO**<br>**WHY not?**<br>***(circle all answers indicated)*** ……….**then Skip to 4A**<br>A. No health workers available<br>B. No trained health workers<br>C. No equipment e.g. machines or beds<br>D. No commodities or supplies<br>E. Against hospital or management policies<br>F. No cases requiring the procedure<br>G. Against the workers morals/ethics
23	Resuscitate newborn with bag and mask	**YES**………………. 22B<br><br>**NO**<br>**WHY not?**<br>***(circle all answers indicated)*** ……….**then Skip to 4A**<br>A. No health workers available<br>B. No trained health workers<br>C. No equipment e.g. machines or beds<br>D. No commodities or supplies<br>E. Against hospital or management policies<br>F. No cases requiring the procedure<br>G. Against the workers morals/ethics
24		**YES**………………. 23B<br><br>**NO**<br>**WHY not?**

	Administer corticosteroids in preterm labour	***(circle all answers indicated)*** ……….**then Skip to 4A**<br>1. No health workers available<br>2. No trained health workers<br>3. No equipment e.g. machines or beds<br>4. No commodities or supplies<br>5. Against hospital or management policies<br>6. No cases requiring the procedure<br>7. Against the workers morals/ethics
25	Provide Kangaroo mother care for preterm or very small babies	**YES**………………. 24B<br><br>**NO**<br>**WHY not?**<br>***(circle all answers indicated)*** ……….**then Skip to 4A**<br>A. No health workers available<br>B. No trained health workers<br>C. No equipment e.g. machines or beds<br>D. No commodities or supplies<br>E. Against hospital or management policies<br>F. No cases requiring the procedure<br>G. Against the workers morals/ethics
26	Provide alternative feeding if baby cannot breastfeed	**YES**………………. 25B<br><br>**NO**<br>**WHY not?**<br>***(circle all answers indicated)*** ……….**then Skip to 4A**<br>A. No health workers available<br>B. No trained health workers<br>C. No equipment e.g. machines or beds<br>D. No commodities or supplies<br>E. Against hospital or management policies<br>F. No cases requiring the procedure<br>G. Against the workers morals/ethics
27	Adminster oxygen to baby	**YES**………………. 26B<br><br>**NO**<br>**WHY not?**<br>***(circle all answers indicated)*** ……….**then Skip to 4A**<br>· No health workers available<br>· No trained health workers<br>· No equipment e.g. machines or beds<br>· No commodities or supplies<br>· Against hospital or management policies<br>· No cases requiring the procedure<br>· Against the workers morals/ethics
28		**YES.**………………. 27B<br>**Where is the primary source of the blood supplied?**<br><br>Central blood bank.......................................1

	Blood Transfusion	The facility has a blood bank........................2<br>Direct transfusion from a family member....3<br>Other (specify)________________________4
		**NO**<br>**WHY not?**<br>***(circle all answers indicated)*** …….**then Skip to 10A**<br>A. No health workers available<br>B. No trained health workers<br>C. No equipment e.g. machines or beds<br>D. No commodities or supplies<br>E. Against hospital or management policies<br>F. No cases requiring the procedure<br>G. Against the workers morals/ethics
29	Surgical procedure to manage abortion complications e.g. laparotomy/ laparoscopy/hysterectomy?	**YES…………………………Skip to 28B**<br><br>**NO**<br>**WHY not?**<br>***(circle all answers indicated)*** …….**Skip to Section 2**<br>A. No health workers available<br>B. No trained health workers<br>C. No equipment e.g. machines or beds<br>D. No commodities or supplies<br>E. Against hospital or management policies<br>F. No cases requiring the procedure<br>G. Against the workers morals/ethics

Name of respondent to this section:____

Position of respondent to this section in facility:________________________________

B. Has your facility done this in the past 3 months?	C. Has your facility done this in the past 12 months?
**YES**……………….skip to 2A<br><br>**NO** ……………….skip 1C<br>**WHY not?**<br>***(circle all answers indicated)***<br>A. No health workers available<br>B. No trained health workers<br>C. No equipment e.g. machines or beds<br>D. No commodities or supplies<br>E. Against hospital or management policies<br>F. No cases requiring the procedure<br>G. Against the workers morals/ethics	**YES**……………….skip to 2A<br><br>**NO**<br>**WHY not?**<br>***(circle all answers indicated)***<br>A. No health workers available<br>B. No trained health workers<br>C. No equipment e.g. machines or beds<br>D. No commodities or supplies<br>E. Against hospital or management policies<br>F. No cases requiring the procedure<br>G. Against the workers morals/ethics
**YES……………….skip to 3A**<br><br>**NO ……………….skip 2C**<br>**WHY not?**<br>***(circle all answers indicated)***<br>1. No health workers available<br>2. No trained health workers<br>3. No equipment e.g. machines or beds<br>4. No commodities or supplies<br>5. Against hospital or management policies<br>6. No cases requiring the procedure<br>7. Against the workers morals/ethics	**YES……………….skip to 3A**<br><br>**NO**<br>**WHY not?**<br>***(circle all answers indicated)***<br>1. No health workers available<br>2. No trained health workers<br>3. No equipment e.g. machines or beds<br>4. No commodities or supplies<br>5. Against hospital or management policies<br>6. No cases requiring the procedure<br>7. Against the workers morals/ethics
**YES**……………….Skip to 4A<br><br>**NO** ……………….Skip to 3C<br>**WHY not?**<br>***(circle all answers indicated)***<br>A. No health workers available<br>B. No trained health workers<br>C. No equipment e.g. machines or beds<br>D. No commodities or supplies<br>E. Against hospital or management policies	**YES**……………….Skip to 4A<br><br>**NO**<br>**WHY not?**<br>***(circle all answers indicated)***<br>A. No health workers available<br>B. No trained health workers<br>C. No equipment e.g. machines or beds<br>D. No commodities or supplies<br>E. Against hospital or management policies

F. No cases requiring the procedure<br>G. Against the workers morals/ethics	F. No cases requiring the procedure<br>G. Against the workers morals/ethics
**YES**……………….Skip to 5A<br><br>**NO**……………….skip to 4C<br>**WHY not?**<br>***(circle all answers indicated)***<br>A. No health workers available<br>B. No trained health workers<br>C. No equipment e.g. machines or beds<br>D. No commodities or supplies<br>E. Against hospital or management policies<br>F. No cases requiring the procedure<br>G. Against the workers morals/ethics	**YES**……………….Skip to 5A<br><br>**NO**<br>**WHY not?**<br>***(circle all answers indicated)***<br>A. No health workers available<br>B. No trained health workers<br>C. No equipment e.g. machines or beds<br>D. No commodities or supplies<br>E. Against hospital or management policies<br>F. No cases requiring the procedure<br>G. Against the workers morals/ethics
**YES**……………….Skip to 6A<br><br>**NO**……………….skip to 5C<br>**WHY not?**<br>***(circle all answers indicated)***<br>1. No health workers available<br>2. No trained health workers<br>3. No equipment e.g. machines or beds<br>4. No commodities or supplies<br>5. Against hospital or management policies<br>6. No cases requiring the procedure<br>**7.** Against the workers morals/ethics	**YES**……………….Skip to 6A<br><br>**NO**<br>**WHY not?**<br>***(circle all answers indicated)***<br>1. No health workers available<br>2. No trained health workers<br>3. No equipment e.g. machines or beds<br>4. No commodities or supplies<br>5. Against hospital or management policies<br>6. No cases requiring the procedure<br>**7.** Against the workers morals/ethics
**YES**……………….Skip to 7A<br><br>**NO**……………….skip to 6C<br>**WHY not?**<br>***(circle all answers indicated)***<br>A. No health workers available<br>B. No trained health workers<br>C. No equipment e.g. machines or beds<br>D. No commodities or supplies<br>E. Against hospital or management policies<br>F. No cases requiring the procedure<br>G. Against the workers morals/ethics	**YES**……………….Skip to 7A<br><br>**NO**<br>**WHY not?**<br>***(circle all answers indicated)***<br>A. No health workers available<br>B. No trained health workers<br>C. No equipment e.g. machines or beds<br>D. No commodities or supplies<br>E. Against hospital or management policies<br>F. No cases requiring the procedure<br>G. Against the workers morals/ethics

**YES**……………….Skip to 9A<br><br>**NO**……………….skip to 8C<br>**WHY not?**<br>***(circle all answers indicated)***<br>A. No health workers available<br>B. No trained health workers<br>C. No equipment e.g. machines or beds<br>D. No commodities or supplies<br>E. Against hospital or management policies<br>F. No cases requiring the procedure<br>G. Against the workers morals/ethics	**YES**……………….Skip to 9A<br><br>**NO**<br>**WHY not?**<br>***(circle all answers indicated)***<br>A. No health workers available<br>B. No trained health workers<br>C. No equipment e.g. machines or beds<br>D. No commodities or supplies<br>E. Against hospital or management policies<br>F. No cases requiring the procedure<br>G. Against the workers morals/ethics
**YES**……………….skip to 10A<br><br>**NO** ……………….skip to 9C<br>**WHY not?**<br>***(circle all answers indicated)***<br>1. No health workers available<br>2. No trained health workers<br>3. No equipment e.g. machines or beds<br>4. No commodities or supplies<br>5. Against hospital or management policies<br>6. No cases requiring the procedure<br>**7.** Against the workers morals/ethics	**YES**……………….skip to 10A<br><br>**NO**<br>**WHY not?**<br>***(circle all answers indicated)***<br>1. No health workers available<br>2. No trained health workers<br>3. No equipment e.g. machines or beds<br>4. No commodities or supplies<br>5. Against hospital or management policies<br>6. No cases requiring the procedure<br>**7.** Against the workers morals/ethics
**YES**……………….skip to 11A<br><br>**NO**……………….Skip to 10C<br>**WHY not?**<br>***(circle all answers indicated)***<br>1. No health workers available<br>2. No trained health workers<br>3. No equipment e.g. machines or beds	**YES**……………….skip to 11A<br><br>**NO**<br>**WHY not?**<br>***(circle all answers indicated)***<br>1. No health workers available<br>2. No trained health workers<br>3. No equipment e.g. machines or beds

4. No commodities or supplies<br>5. Against hospital or management policies<br>6. No cases requiring the procedure<br>**7.** Against the workers morals/ethics	4. No commodities or supplies<br>5. Against hospital or management policies<br>6. No cases requiring the procedure<br>**7.** Against the workers morals/ethics
**YES**……………….Skip to 12A<br><br>**NO** ……………….Skip to 11C<br>**WHY not?**<br>***(circle all answers indicated)***<br>A. No health workers available<br>B. No trained health workers<br>C. No equipment e.g. machines or beds<br>D. No commodities or supplies<br>E. Against hospital or management policies<br>F. No cases requiring the procedure<br>G. Against the workers morals/ethics	**YES**……………….Skip to 12A<br><br>**NO**<br>**WHY not?**<br>***(circle all answers indicated)***<br>A. No health workers available<br>B. No trained health workers<br>C. No equipment e.g. machines or beds<br>D. No commodities or supplies<br>E. Against hospital or management policies<br>F. No cases requiring the procedure<br>G. Against the workers morals/ethics
**YES**……………….Skip to 13A<br><br>**NO**……………….Skip to12C<br>**WHY not?**<br>***(circle all answers indicated)***<br>A. No health workers available<br>B. No trained health workers<br>C. No equipment e.g. machines or beds<br>D. No commodities or supplies<br>E. Against hospital or management policies<br>F. No cases requiring the procedure	**YES**……………….Skip to 13A<br><br>**NO**<br>**WHY not?**<br>***(circle all answers indicated)***<br>1. No health workers available<br>2. No trained health workers<br>3. No equipment e.g. machines or beds<br>4. No commodities or supplies<br>5. Against hospital or management policies<br>6. No cases requiring the procedure

G. Against the workers morals/ethics	Against the workers morals/ethics
**YES**………………Skip to 14A<br><br>**NO** ………………Skip to 13C<br>**WHY not?**<br>***(circle all answers indicated)***<br>A. No health workers available<br>B. No trained health workers<br>C. No equipment e.g. machines or beds<br>D. No commodities or supplies<br>E. Against hospital or management policies<br>F. No cases requiring the procedure<br>G. Against the workers morals/ethics	**YES**………………Skip to 14A<br><br>**NO**<br>**WHY not?**<br>***(circle all answers indicated)***<br>1. No health workers available<br>2. No trained health workers<br>3. No equipment e.g. machines or beds<br>4. No commodities or supplies<br>5. Against hospital or management policies<br>6. No cases requiring the procedure<br>7. Against the workers morals/ethics
**YES**………………Skip to 15A<br><br>**NO………………..Skip to 14C**<br>**WHY not?**<br>***(circle all answers indicated)***<br>A. No health workers available	**YES**………………Skip to 15A<br><br>**NO**<br>**WHY not?**<br>***(circle all answers indicated)***<br>1. No health workers available

B. No trained health workers<br>C. No equipment e.g. machines or beds<br>D. No commodities or supplies<br>E. Against hospital or management policies<br>F. No cases requiring the procedure<br>G. Against the workers morals/ethics	2. No trained health workers<br>3. No equipment e.g. machines or beds<br>4. No commodities or supplies<br>5. Against hospital or management policies<br>6. No cases requiring the procedure<br>**7.** Against the workers morals/ethics
**YES**……………….Skip to 16A<br><br>**NO** ……………….Skip to 15C<br>**WHY not?**<br>***(circle all answers indicated)***<br>A. No health workers available<br>B. No trained health workers<br>C. No equipment e.g. machines or beds<br>D. No commodities or supplies<br>E. Against hospital or management policies<br>F. No cases requiring the procedure<br>G. Against the workers morals/ethics	**YES**……………….Skip to 16A<br><br>**NO**<br>**WHY not?**<br>***(circle all answers indicated)***<br>1. No health workers available<br>2. No trained health workers<br>3. No equipment e.g. machines or beds<br>4. No commodities or supplies<br>5. Against hospital or management policies<br>6. No cases requiring the procedure<br>**7.** Against the workers morals/ethics
**YES**……………….skip to 17A<br>**NO** ……………….Skip to 176C<br><br>**WHY not?**<br>***(circle all answers indicated)***<br>1. No health workers available<br>2. No trained health workers<br>3. No equipment e.g. machines or beds<br>4. No commodities or supplies	**YES**……………….skip to 17A<br>**NO**<br><br>**WHY not?**<br>***(circle all answers indicated)***<br>1. No health workers available<br>2. No trained health workers<br>3. No equipment e.g. machines or beds<br>4. No commodities or supplies

5. Against hospital or management policies<br>6. No cases requiring the procedure<br>**7.** Against the workers morals/ethics	5. Against hospital or management policies<br>6. No cases requiring the procedure<br>**7.** Against the workers morals/ethics
**YES**……………….skip to 18A<br>**NO** ……………….skip to 17C<br><br>**WHY not?**<br>***(circle all answers indicated)***<br>A. No health workers available<br>B. No trained health workers<br>C. No equipment e.g. machines or beds<br>D. No commodities or supplies<br>E. Against hospital or management policies<br>F. No cases requiring the procedure<br>G. Against the workers morals/ethics	**YES**……………….skip to 18A<br>**NO**<br><br>**WHY not?**<br>***(circle all answers indicated)***<br>1. No health workers available<br>2. No trained health workers<br>3. No equipment e.g. machines or beds<br>4. No commodities or supplies<br>5. Against hospital or management policies<br>6. No cases requiring the procedure<br>**7.** Against the workers morals/ethics

**YES**……………….Skip to 22A<br><br>**NO**……………….skip to 21C<br>**WHY not?**<br>***(circle all answers indicated)***<br>1. No health workers available<br>2. No trained health workers<br>3. No equipment e.g. machines or beds<br>4. No commodities or supplies<br>5. Against hospital or management policies<br>6. No cases requiring the procedure<br>7. Against the workers morals/ethics	**YES**……………….Skip to 22A<br><br>**NO**<br>**WHY not?**<br>***(circle all answers indicated)***<br>1. No health workers available<br>2. No trained health workers<br>3. No equipment e.g. machines or beds<br>4. No commodities or supplies<br>5. Against hospital or management policies<br>6. No cases requiring the procedure<br>7. Against the workers morals/ethics
**YES**……………….Skip to 23A<br><br>**NO**……………….skip to 22C<br>**WHY not?**<br>***(circle all answers indicated)***<br>A. No health workers available<br>B. No trained health workers<br>C. No equipment e.g. machines or beds<br>D. No commodities or supplies<br>E. Against hospital or management policies<br>F. No cases requiring the procedure<br>G. Against the workers morals/ethics	**YES**……………….Skip to 23A<br><br>**NO**<br>**WHY not?**<br>***(circle all answers indicated)***<br>A. No health workers available<br>B. No trained health workers<br>C. No equipment e.g. machines or beds<br>D. No commodities or supplies<br>E. Against hospital or management policies<br>F. No cases requiring the procedure<br>G. Against the workers morals/ethics
**YES**……………….Skip to 24A<br><br>**NO**……………….skip to 23C<br>**WHY not?**	**YES**……………….Skip to 24A<br><br>**NO**<br>**WHY not?**

(circle all answers indicated)<br>1. No health workers available<br>2. No trained health workers<br>3. No equipment e.g. machines or beds<br>4. No commodities or supplies<br>5. Against hospital or management policies<br>6. No cases requiring the procedure<br>7. Against the workers morals/ethics	***(circle all answers indicated)***<br>1. No health workers available<br>2. No trained health workers<br>3. No equipment e.g. machines or beds<br>4. No commodities or supplies<br>5. Against hospital or management policies<br>6. No cases requiring the procedure<br>7. Against the workers morals/ethics
**YES**……………….Skip to 25A<br><br>**NO**……………….skip to 24C<br>**WHY not?**<br>***(circle all answers indicated)***<br>A. No health workers available<br>B. No trained health workers<br>C. No equipment e.g. machines or beds<br>D. No commodities or supplies<br>E. Against hospital or management policies<br>F. No cases requiring the procedure<br>G. Against the workers morals/ethics	**YES**……………….Skip to 25A<br><br>**NO**<br>**WHY not?**<br>***(circle all answers indicated)***<br>A. No health workers available<br>B. No trained health workers<br>C. No equipment e.g. machines or beds<br>D. No commodities or supplies<br>E. Against hospital or management policies<br>F. No cases requiring the procedure<br>G. Against the workers morals/ethics
**YES**……………….Skip to 26A<br><br>**NO**……………….skip to 25C<br>**WHY not?**<br>***(circle all answers indicated)***<br>· No health workers available<br>· No trained health workers<br>· No equipment e.g. machines or beds<br>· No commodities or supplies<br>· Against hospital or management policies<br>· No cases requiring the procedure<br>· Against the workers morals/ethics	**YES**……………….Skip to 26A<br><br>**NO**<br>**WHY not?**<br>***(circle all answers indicated)***<br>· No health workers available<br>· No trained health workers<br>· No equipment e.g. machines or beds<br>· No commodities or supplies<br>· Against hospital or management policies<br>· No cases requiring the procedure<br>· Against the workers morals/ethics
**YES**……………….Skip to 27A<br><br>**NO**……………….skip to 26C<br>**WHY not?**<br>***(circle all answers indicated)***<br>· No health workers available<br>· No trained health workers<br>· No equipment e.g. machines or beds<br>· No commodities or supplies<br>· Against hospital or management policies<br>· No cases requiring the procedure<br>· Against the workers morals/ethics	**YES**……………….Skip to 27A<br><br>**NO**<br>**WHY not?**<br>***(circle all answers indicated)***<br>· No health workers available<br>· No trained health workers<br>· No equipment e.g. machines or beds<br>· No commodities or supplies<br>· Against hospital or management policies<br>· No cases requiring the procedure<br>· Against the workers morals/ethics
**YES**……………….Skip to 28A<br><br>**NO** ……………….Skip to 27C<br>**WHY not?**	**YES**……………….Skip to 28A<br><br>**NO**<br>**WHY not?**

(circle all answers indicated)<br>A. No health workers available<br>B. No trained health workers<br>C. No equipment e.g. machines or beds<br>D. No commodities or supplies<br>E. Against hospital or management policies<br>F. No cases requiring the procedure<br>G. Against the workers morals/ethics	***(circle all answers indicated)***<br>A. No health workers available<br>B. No trained health workers<br>C. No equipment e.g. machines or beds<br>D. No commodities or supplies<br>E. Against hospital or management policies<br>F. No cases requiring the procedure<br>G. Against the workers morals/ethics
**YES**………………Skip to 29A<br><br>**NO** ………………Skip to 28C<br>**WHY not?**<br>***(circle all answers indicated)***<br>A. No health workers available<br>B. No trained health workers<br>C. No equipment e.g. machines or beds<br>D. No commodities or supplies<br>E. Against hospital or management policies<br>F. No cases requiring the procedure<br>G. Against the workers morals/ethics	**YES**………………Skip to 29A2<br><br>**NO**<br>**WHY not?**<br>***(circle all answers indicated)***<br>A. No health workers available<br>B. No trained health workers<br>C. No equipment e.g. machines or beds<br>D. No commodities or supplies<br>E. Against hospital or management policies<br>F. No cases requiring the procedure<br>G. Against the workers morals/ethics

Section 2	
A. Have some of the staff registered at this facility been trained on comprehensive abortion care?	1 Yes
B. What cadre of staff underwent training *(Circle appropriate response)*	Yes
a. Doctors	1
b. Clinical officers	1
c. Nurses	1
d. Other (please specify)...	1
C. Are there any trained health providers who provide comprehensive abortion care here but do not work full-time at this facility?	1 Yes
D. Has any organization provided any equipment or medical commodities to this facility for comprehensive abortion care?	1 Yes
E. What kind of equipment/commodities have they provided:	______________<br>______________
F. When last were such commodities supplied? *(Select appropriate response)*	A In the l<br>B In the l<br>C In the<br>D 1 year<br>E Over 1
G. Have some of the staff registered at this facility been trained by any other NGO's or organizations on comprehensive abortion care?	1 Yes
Name of respondent to this section	______________
Position of respondent to this section in facility	______________

2 No (Then skip to Question 3)

No

2

2

2

2

2 No

2 No(End interview here)

____

____

last 1 month

last 3 months

last 6 months

ago

1 year ago.

2 No

____

____

Q	A. Can your facility perform this procedure?	
	Does this facility offer HIV counseling and testing services?	**YES…………………………Skip to 29B**<br><br>**NO**<br>**WHY not?**<br>***(circle all answers indicated)*** …….**Skip to Section 2**<br>A. No health workers available<br>B. No trained health workers<br>C. No equipment e.g. machines or beds<br>D. No commodities or supplies<br>E. Against hospital or management policies<br>F. No cases requiring the procedure<br>G. Against the workers morals/ethics
	Hepatitis B screening?	**YES…………………………Skip to 30B**<br><br>**NO**<br>**WHY not?**<br>***(circle all answers indicated)*** …….**Skip to Section 2**<br>A. No health workers available<br>B. No trained health workers<br>C. No equipment e.g. machines or beds<br>D. No commodities or supplies<br>E. Against hospital or management policies<br>F. No cases requiring the procedure<br>G. Against the workers morals/ethics
	STI (Chlamydia, Gonorrhea, Trichomona vaginalis, and Syphilis) testing are you asking	**YES…………………………Skip to 31B**<br><br>**NO**<br>**WHY not?**<br>***(circle all answers indicated)*** …….**Skip to Section 2**<br>A. No health workers available<br>B. No trained health workers<br>C. No equipment e.g. machines or beds<br>D. No commodities or supplies

	about all? Or any of the above	E. Against hospital or management policies<br>F. No cases requiring the procedure<br>G. Against the workers morals/ethics
	**Name of respondent to this section:**___________________<br><br>**Position of respondent to this section in facility:**_________	

LABORATORY PROCEDURES

Is there a laboratory in this facility- Y/N

B. Has your facility done this in the past 3 months?	C. Has your facility done this in the past 12 months?
**YES**……………….Skip to 30A<br><br>**NO** ……………….Skip to 29C<br>**WHY not?**<br>***(circle all answers indicated)***<br>A. No health workers available<br>B. No trained health workers<br>C. No equipment e.g. machines or beds<br>D. No commodities or supplies<br>E. Against hospital or management policies<br>F. No cases requiring the procedure<br>G. Against the workers morals/ethics	**YES**……………….Skip to 30A<br><br>**NO**<br>**WHY not?**<br>***(circle all answers indicated)***<br>A. No health workers available<br>B. No trained health workers<br>C. No equipment e.g. machines or beds<br>D. No commodities or supplies<br>E. Against hospital or management policies<br>F. No cases requiring the procedure<br>G. Against the workers morals/ethics
**YES**……………….Skip to 31A<br><br>**NO** ……………….Skip to 30C<br>**WHY not?**<br>***(circle all answers indicated)***<br>A. No health workers available<br>B. No trained health workers<br>C. No equipment e.g. machines or beds<br>D. No commodities or supplies<br>E. Against hospital or management policies<br>F. No cases requiring the procedure<br>G. Against the workers morals/ethics	**YES**……………….Skip to 31A<br><br>**NO**<br>**WHY not?**<br>***(circle all answers indicated)***<br>A. No health workers available<br>B. No trained health workers<br>C. No equipment e.g. machines or beds<br>D. No commodities or supplies<br>E. Against hospital or management policies<br>F. No cases requiring the procedure<br>G. Against the workers morals/ethics
**YES**……………….Skip to Section 2<br><br>**NO** ……………….Skip to 31C<br>**WHY not?**<br>***(circle all answers indicated)***<br>1. No health workers available<br>2. No trained health workers<br>3. No equipment e.g. machines or beds<br>4. No commodities or supplies	**YES**……………….Skip to Section 2<br><br>**NO**<br>**WHY not?**<br>***(circle all answers indicated)***<br>A. No health workers available<br>B. No trained health workers<br>C. No equipment e.g. machines or beds<br>D. No commodities or supplies

5. Against hospital or management policies<br>6. No cases requiring the procedure<br>7. Against the workers morals/ethics	E. Against hospital or management policies<br>F. No cases requiring the procedure<br>G. Against the workers morals/ethics

____

____

		HOSPITAL EQUIPMENT	
	**Section 2**		
	**I would like to know if the following items are available in this service area today. For each equipment or item, please tell me if it is available today and functioning?**	**Reported Available(Circle appropriate response)**	
1	**Private room for examining /counselling women and performing reproductive health procedures**	**Yes**	**No**
2	**Water source**	**Yes**	**No**
3	**Electric autoclave/non-electric autoclave/Electric dry heat sterilizer/ Electric boiler or steamer/ Non-electric pot with cover for boiling/steam /**	**Yes**	**No**
4	**Vehicle (ambulance or other vehicle) to transfer referred patients**	**Yes**	**No**
5	**Is fuel for the ambulance or other emergency vehicle available today?**	**Yes**	**No**
6	**Functional Landline/Mobile phone supported by the facility**	**Yes**	**No**
7	**Does your facility have electricity from any source (e.g. electricity grid, generator, solar, or other) including for stand-alone devices (EPI cold chain)?**	**Yes**	**No**
8	**Is there a toilet (latrine) on premises in *functioning condition* that is accessible for general outpatient client use? IF YES: What type of toilet? IF MULTIPLE TOILETS ARE AVAILABLE, CONSIDER THE MOST MODERN TYPE**	**Yes**	**No**
9	**Ultrasound**	**Yes**	**No**
10	**Examination light/ Light source (flashlight acceptable)**	**Yes**	**No**
11	**Delivery bed**	**Yes**	**No**
12	**Stethoscope**	**Yes**	**No**
13	**Digital or manual sphygmomanometer**	**Yes**	**No**

14	IV fluid (neonatal giving) set/umbilical catheter	Yes	No
15	IV fluid giving set (adult)	Yes	No
16	Needles and syringes	Yes	No
17	Disposable latex gloves	Yes	No
18	Hand-washing soap/liquid soap	Yes	No
19	Alcohol based hand rub	Yes	No
20	Sterile theater gowns	Yes	No
21	Delivery pack	Yes	No
22	Vacuum extractor with different size cups	Yes	No
23	Obstetric forceps	Yes	No
24	Vaginal speculum (Sims, Cusco)	Yes	No
25	Uristix (dip stick for protein in urine)	Yes	No
26	Watch or clock with second hand that can be easily seen	Yes	No
27	Blood sugar testing sticks	Yes	No
28	Cord clamp	Yes	No
29	4% CHORHEXIDINE SOLUTION (UMBILICAL CORD	Yes	No
30	Infant weighing scale	Yes	No
31	Towels or cloth for newborn	Yes	No
32	Small cup for breast milk expression	Yes	No
33	Mucus extractor	Yes	No
34	Resuscitation table with heat source for newborn	Yes	No
35	Incubator	Yes	No
36	Infant face masks, sizes 0, 1, 2	Yes	No

37	Ambu (ventilatory) bag	Yes	No
38	Suction catheter, 10, 12 Ch	Yes	No
39	Suction aspirator (operated by foot or electrically)	Yes	No
40	Infant laryngoscope with spare bulb and batteries	Yes	No
41	Endotracheal tubes, 3.5, 3.0	Yes	No
42	Disposable uncuffed tracheal tubes, sizes 2.0 to 3.5	Yes	No
43	Mucus trap for suction	Yes	No
44	Vacuum aspirator kit/syringes	Yes	No
	**Dilatation and Curettage (D&C) kit**		
45	Vaginal speculum, (Sims)	Yes	No
46	Sponge (ring) forceps	Yes	No
47	Dissecting forceps, serrated jaws 250mm S/S	Yes	No
48	Towel clip	Yes	No
49	Ovum forceps	Yes	No
50	Uterine forceps, 3x4 teeth, curved, S/S	Yes	No
51	Uterine forceps, S/S	Yes	No
52	Uterine dilators, sizes 13-27	Yes	No
53	Sharp uterine curettes	Yes	No
54	Blunt uterine curettes	Yes	No
55	Uterine sound	Yes	No
	Functional operating theatre	Yes	No (if no sk
	**Laparotomy/Caesarean kit**		
		Yes	No

56	Stainless steel instrument tray with cover	Yes	No
57	Towel clips	Yes	No
58	Sponge (ring) forceps	Yes	No
59	Straight artery forceps	Yes	No
60	Uterine hemostasis forceps	Yes	No
61	Needle holder	Yes	No
62	Surgical knife handle, No. 3 or 4	Yes	No
63	Surgical knife blades	Yes	No
64	Triangular point suture needles, 7.3cm/size 6	Yes	No
65	Round-bodied needles, No. 12/size 6	Yes	No
66	Abdominal retractor	Yes	No
67	Abdominal retractors, double-ended	Yes	No
68	Operating scissors, curved	Yes	No
69	Operating scissors, straight	Yes	No
70	Scissors, straight,	Yes	No
71	Suction nozzle	Yes	No
72	Suction tube, 22.5cm, 23 French gauge	Yes	No
73	Intestinal clamps, curved, 22.5cm	Yes	No
74	Intestinal clamps, straight, 22.5cm	Yes	No
75	Dressing (tissue) forceps, non-toothed,	Yes	No
76	Sutures (different sizes and types)	Yes	No
77	Mini-laparotomy kit	Yes	No
	Anaesthesia equipment	Yes	No
78	Anesthetic face masks	Yes	No

79	Oropharyngeal airways	Yes	No
80	Laryngoscopes with spare bulbs and batteries	Yes	No
81	Endotracheal tubes with cuffs, 8mm	Yes	No
82	Endotracheal tubes with cuffs, 10mm	Yes	No
83	Intubating forceps	Yes	No
84	Endotracheal tube connectors, plastic, 15 mm (connect directly to breathing valve; three for each tube size)	Yes	No
85	Spinal needles, 18 gauge to 25 gauge	Yes	No
86	Suction aspirator, foot-operated	Yes	No
87	Suction aspirator, electric	Yes	No
88	Anesthetic vaporizers (draw-over system)	Yes	No
89	Oxygen cylinders with manometer and flowmeter (low flow) tubes and connectors	Yes	No
90	Cannula and Trochar for implant	Yes	No

Name of respondent to this section: ____________________

Position of respondent to this section in facility: ____________________

Observed (Circle appropriate response)		Functioning (Mark “X” in the appropriate box)		
		Yes	No	DON’T KNOW
Yes	No			
Yes	No			
Yes	No			
Yes	No			
Yes	No			
Yes	No			
Yes	No			
Yes	No			
Yes	No			
Yes	No			
Yes	No			
Yes	No			
Yes	No			

Yes	No			
Yes	No			
Yes	No			
Yes	No			
Yes	No			
Yes	No			
Yes	No			
Yes	No			
Yes	No			
Yes	No			
Yes	No			
Yes	No			
Yes	No			

Yes	No			
Yes	No			
Yes	No			

Yes	No			
Yes	No			
Yes	No			
Yes	No			
Yes	No			
Yes	No			
Yes	No			

Yes	No	
Yes	No	
Yes	No	
Yes	No	
Yes	No	
Yes	No	
Yes	No	
Yes	No	
Yes	No	
Yes	No	
Yes	No	
Yes	No	
Yes	No	
Yes	No	
Yes	No	
Yes	No	
Yes	No	
Yes	No	
Yes	No	

:ip to section 3)

Yes		

Yes	No			
Yes	No			
Yes	No			
Yes	No			
Yes	No			
Yes	No			
Yes	No			
Yes	No			
Yes	No			
Yes	No			
Yes	No			
Yes	No			
Yes	No			
Yes	No			
Yes	No			
Yes	No			
Yes	No			
Yes	No			
Yes	No			
Yes	No			
Yes	No			
Yes	No			
Yes	No			
Yes	No			

Yes	No			
Yes	No			
Yes	No			
Yes	No			
Yes	No			
Yes	No			
Yes	No			
Yes	No			
Yes	No			
Yes	No			
Yes	No			
Yes	No			

Section 3				
	Are any of the following medicines and commodities available in this service site today?	1. Never available		2. Not a
1	Mifepristone and misoprostol combination..	Yes	No	Yes
2	Misoprostol alone	Yes	No	Yes
3	Other abortifacient (specify)....	Yes	No	Yes
4	Injectable uterotonic (e.g. oxytocin)	Yes	No	Yes
5	Magnesium sulphate injection	Yes	No	Yes
6	Do you have any other anticonvulsants such as- diazepam injection, Phenobarbital (injection), Phenytoin (Diphenylhydantoin)	Yes	No	Yes
7	Do you have steroids usually administered to women who have preterm labor such as-Betamethasone injection, Dexamethasone injection	Yes	No	Yes
8	Do you have any of these antibiotics-Amoxicillin, ampicillin, azithromycin, Cephazoline sodium, Co-trimoxazole cap/tab (Oral antibiotic), Cefixime, Ceftriaxone, Cefotaxime injection (for newborn), Chloramphenicol (injection), Ciprofloxacin, Clindamycin, Cloxacillin sodium, Doxycycline, Erythromycin, Metronidazole (injection), Penicillin G (Benzyl), Trimethoprim/sulfamethoxazole, Tetracycline	Yes	No	Yes
9	Benzathine benzyl penicillin powder for injection	Yes	No	Yes
10	Oral flucloxacillin (for newborn)	Yes	No	Yes
11	Gentamicin (injection)	Yes	No	Yes
12	Tetracycline eye ointment/drops	Yes	No	Yes
13	Halothane	Yes	No	Yes
14	Ketamine	Yes	No	Yes

15	**Lignocaine/Lidocaine 2% or 1%**	Yes	No	Yes
16	**Dextrose**	Yes	No	Yes
17	**Dextran**	Yes	No	Yes
18	**Glucose infusion 5%**	Yes	No	Yes
19	**Normal saline**	Yes	No	Yes
20	**Ringer’s lactate**	Yes	No	Yes
21	**Nevirapine (for mother)**	Yes	No	Yes
22	**Nevirapine (for newborn)**	Yes	No	Yes
23	**Post-HIV exposure prophylactic treatment**	Yes	No	Yes
24	**Combined ARVs for mother**	Yes	No	Yes
25	**Combined ARVs for newborn**	Yes	No	Yes
26	**Hepatitis B immunization**	Yes	No	Yes
27	**Combined oral contraceptive pills**	Yes	No	Yes
28	**Progestin-only contraceptive pills**	Yes	No	Yes
29	**Combined injectable contraceptives**	Yes	No	Yes
30	**Progestin-only injectable contraceptives (Depo)**	Yes	No	Yes
31	**Male condoms**	Yes	No	Yes
32	**Female condoms**	Yes	No	Yes
33	**Intrauterine contraceptive device (IUCD)**	Yes	No	Yes
34	**Implant**	Yes	No	Yes
35	**Emergency contraceptive pills**	Yes	No	Yes
36	**Syphilis rapid test kit**	Yes	No	Yes
37	**HIV rapid test kit**	Yes	No	Yes
38	**Urine pregnancy test kit**	Yes	No	Yes

Time Interview ended

H	H	:	M	A/P

A. Duration of time the interview took: [ ] Hours [ ] Minutes

Interview information sheet- TO BE FILLED IN IMMEDIATELY AFTER THE INTERVIEW IS COMPLETED

vailable today	3. Reported available	4. Obs 1+ unexpired	5. Obs all expired	Obs DK
No				
No				
No				
No				
No				
No				
No				
No				
No				
No				
No				
No				
No				
No				

No				
No				
No				
No				
No				
No				
No				
No				
No				
No				
No				
No				
No				
No				
No				
No				
No				
No				
No				
No				
No				
No				
No				
No				

M

s
